# Role of Cytokines and Chemokines in NSCLC Immune Navigation and Proliferation

**DOI:** 10.1155/2021/5563746

**Published:** 2021-07-16

**Authors:** Sowmya Ramachandran, Amit K. Verma, Kapil Dev, Yamini Goyal, Deepti Bhatt, Mohammed A. Alsahli, Arshad Husain Rahmani, Ahmad Almatroudi, Saleh A. Almatroodi, Faris Alrumaihi, Naushad Ahmad Khan

**Affiliations:** ^1^School of Pharmaceutical Sciences, Universiti Sains Malaysia, Main Campus, Penang, Malaysia; ^2^Department of Biotechnology, Jamia Millia Islamia, New Delhi, India; ^3^Department of Medical Laboratories, College of Applied Medical Sciences, Qassim University, Buraidah, Saudi Arabia; ^4^Department of Biochemistry, Faculty of Medical Sciences, Alatoo International University, Bishkek, Kyrgyzstan; ^5^Department of Trauma and Surgery, Hamad Medical Corporation, Doha, Qatar

## Abstract

With over a million deaths every year around the world, lung cancer is found to be the most recurrent cancer among all types. Nonsmall cell lung carcinoma (NSCLC) amounts to about 85% of the entire cases. The other 15% owes it to small cell lung carcinoma (SCLC). Despite decades of research, the prognosis for NSCLC patients is poorly understood with treatment options limited. First, this article emphasises on the part that tumour microenvironment (TME) and its constituents play in lung cancer progression. This review also highlights the inflammatory (pro- or anti-) roles of different cytokines (ILs, TGF-*β*, and TNF-*α*) and chemokine (CC, CXC, C, and CX3C) families in the lung TME, provoking tumour growth and subsequent metastasis. The write-up also pinpoints recent developments in the field of chemokine biology. Additionally, it covers the role of extracellular vesicles (EVs), as alternate carriers of cytokines and chemokines. This allows the cytokines/chemokines to modulate the EVs for their secretion, trafficking, and aid in cancer proliferation. In the end, this review also stresses on the role of these factors as prognostic biomarkers for lung immunotherapy, apart from focusing on inflammatory actions of these chemoattractants.

## 1. Introduction

Cancer, largely a result of random mutations, goes through a multistep process of carcinogenesis. During the course, several genetic modifications pile up. This ultimately leads to unusual and dissolute cell growth, in most cases, giving rise to the malignant phenotype [1]. Among all types of cancers, lung cancer is not only most common but also poses a grave danger, leading to deaths predominantly among males followed by female patients the world over. Lung cancer, as we know, is heterogeneous and what contributes to its occurrence, explosion, and metastasis are the factors like genetic mutations, environmental, and individual habits. Many genes with specific and intricate cellular pathways are said to play a major part in such processes [2]. Because initial-stage detection of lung cancer is as low as 16%, the survival rate for five years stands at 5% for metastatic lung cancer, and this percentage is significantly lower than that of prostate, breast, and colon cancers [3]. The plausible reason for this could be the lack of effective treatments, despite advances in identifying the key mutations that lead to the progress of molecular-targeted treatments. The majority of lung cancer patients are diagnosed when they have symptoms and are in an advanced stage of the disease, and curative therapy is no longer a choice. Long sought after has been a reliable screening test for early detection with the goal of lowering lung cancer mortality. Potential screening tests include sputum cytology, chest radiography, and computed tomography (CT) scan. Low-dose CT (LDCT) screening reduced mortality by 20% in the National Lung Screening Trial (NLST), and recommendations also recommend annual LDCT screening for those at high risk. Screening is being implemented with the aim of seeing the benefits in clinical practice rather than in a research study environment. Management of false positives, expense, incidental findings, radiation exposure, and overdiagnosis are all concerns. In order to enhance lung cancer patient outcomes, studies are continuing to examine LDCT screening and the use of biomarkers in risk assessment and diagnosis [4].

Unfortunately, the resistance mechanisms of cancer cells have worked overtime to blunt the efficacy of the treatments [5]. For proper lung cancer detection, criteria with pathological basis have been identified by the World Health Organization (WHO). Biopsy through bronchoscopy to identify the morphology of squamous cell carcinoma or adenocarcinoma is considered as the gold standard when it comes to confirmation of lung cancer [6, 7]. When no specific morphological proof is found, the tumour is categorised as NSCLC, which has further subdivisions, and they are based on various attributes like mucin staining, molecular data-based marker examination, and immunohistochemistry (IHC) [8].

In 1878, threatening lung tumours addressed just 1 percent of all malignancies visible during autopsies; these days, they are the main source of disease mortality, with 1∙37 million passings universally every year. Adjuvant chemotherapy expands 5-year endurance by just 5%, and medical procedure like surgeries stays the most ideal choice for accomplishing long haul abatement. New remedial procedures are hence excitedly anticipated. Directed treatments dependent on subatomic changes in subgroups of cellular breakdown in the lungs have effectively furnished some clinical advantage in patients with transformed epidermal development factor receptor (EGFR) or echinoderm microtubule-related protein-like 4-anaplastic lymphoma kinase (EML4-ALK) revision. Immunotherapy that means to invigorate the safe framework is another alternative for treating malignant growth, and there has been reestablished revenue in this methodology since 2011, when the US Food and Medication Organization endorsed ipilimumab for metastatic melanoma and sipuleucel-T immunization for asymptomatic, metastatic, castrate-resistant prostate cancer [9].

### 1.1. Lung Cancer and Its Types

This is categorised into nonsmall cell lung carcinoma (NSCLC), accounting for 85%, and small cell lung carcinoma (SCLC), the remaining 15% of cases [10]. Based on histology, NSCLC has three subdivisions which include adenocarcinoma (ADC), large-cell lung cancer, and squamous cell carcinoma (SCC). NSCLC, which is not gendered and the most common form, is noticed in both smokers and nonsmokers with 40% of them being ADC victims. The origin of ADC is found in type II alveolar cells of lungs' exterior hiding mucus [11]. The other subdivision, SCC, found in the middle of the lungs arising in flat squamous cells laden interior of airways, accounts for about 25 to 30% owing mainly to cigarette smoking [12]. However, large cell (indistinguishable) carcinoma—it can be traced to any part of the lung and spreads quickly—is prevalent in 10 to 15% of cases [8]. Though surgical resection is very much in vogue to treat patients of all types of NSCLC, conventional treatments like radiotherapy and multimodal neoadjuvant chemotherapy are followed [13].

But then, the survival of patients is more often than not associated with the stage in which they are. The survival rate drops as the stage progresses, from over 80% in stage I to about 5% in stage IV [14]. Several cases of asymptomatic cancer patients, during routine clinical practice, are not diagnosed at the right time. But by the time when the subsequent results come in for diagnostic purposes, nearly 70% of NSCLC patients have reached an advanced stage, either locally or have metastasised to other organs [15]. In other words, a possible solution to the survival of lung cancer patients could be an early-warning indicator, the need of the hour. Inflammation is an important part of the specific response of the immune system to harmful incitement. There are numerous research proofs about the role inflammation plays in tumorigenesis [16].

Of the approximate 1.8 million cases each year, the death toll is approximately 1.6 million. Improvements in diagnosis and various treatments notwithstanding, over 50% of patients do not survive more than a year. This curbs the usual 5-year survival percentage to just about 18%! It can be attributed to the hostile and metastatic contrivances of lung cancer with no clarity [17]. This is despite past studies proving that many genes work in unison to cause lung cancer. Hence, understanding the molecular mechanisms confined to the pathogenesis of NSCLC apart from improving diagnosis and therapy for lung cancer patients is imperative [18].

The disease being multifaceted, it acquires genetic and epigenetic variations. These changes tend to control differentiation, proliferation, invasion, and metastasis of tumours. Surgery, considered the most effective treatment for NSCLC in the initial stage, about 70 to 80% of patients are not convinced about it essentially because of locoregional tumour extension, extrathoracic spread, or poor physical and functional condition when diagnosed. Therefore, patients tend to adopt decisive action of radio or chemotherapy separately for better prognosis [19]. Alternatively, the neoadjuvant approach is also followed by surgical resection. Radiotherapy (RT) does play a vital role to help treat lung cancer [20]. Several studies have proved that about 60% of patients rely on RT at the early stage of the ailment—about 44% during diagnosis and about 16% during growth or recurrence [21]. With two-thirds having local or metastatic cancer during diagnosis, chemotherapy is considered a better option to treat lung cancer [22]. The FDA-permitted treatments have mostly aimed at processes like angiogenesis along with a focus on the role of immune checkpoints in describing lung cancer's pathological and physiological attributes of the tumour microenvironment and prognosis-related attributes [2].

### 1.2. Tumour Microenvironment and Its Components—Its Role in Aiding NSCLC Metastasis

The immune system framework comprises an integrated cells' network (lymphocytes, macrophages, dendritic cells, and natural killer cells) that convey through cell-to-cell contact or through surface microparticles and different mediators (cytokines and chemokines). Innate immunity, interceded by macrophages, neutrophils, and NK cells, ordinarily work ahead of schedule over the span of an insusceptible reaction and include a restricted arrangement of receptors. On the other hand, adaptive immunity is constrained by T lymphocytes (CD4+ and CD8+ T cells) and B cells. Initiation of these cells relies upon the presentation of antigens by dendritic cells; B cells are enacted by antigens introduced in their local structure, while CD8+ and CD4+ lymphocytes perceive antigens prepared into peptides complexed with MHC class I and II particles, respectively. Adaptive immune system requires a few days to be viable and instigates robust memory through germinal gene arrangements that create a huge collection of T- and B-cell receptors that can specifically perceive a scope of targets. The cytokine profile delivered by immune cells decides the response of the immune system: TH1 cytokines (e.g., IL-2, IFN-*γ*, and tumour necrosis factor *α* (TNF*α*)) favour cell-mediated immunity; TH2 cytokines (e.g., IL-4, 5, 10, and 13) are significant for humoral immunity and allergy; and TH17 cytokines (e.g., IL-17, 22, and granulocyte-colony stimulating factor (G-CSF)) lead to an inflammatory response. Defensive long-haul resistance against cancer, fit for counteracting chronic inflammation, appears to depend on effector and focal memory T helper type 1 (Th1) and T killer type 1 (Tc1) cells, instead of on humoral immune response. The last effectors of cancer-protective immune response may or probably will not be the last foragers, in that T cells or NK cells can kill tumour cells by direct contact through TNF-receptor family members (e.g., FAS and TRAIL) or through degranulation of perforin and granzymes. Similarly, the last effectors can deliver Th1 cytokines that reprogramme tumour-associated M2 macrophages, which discharge arginase and inducible nitric oxide synthase, into M1 killer cells that produce IL-12 and TNF*α*. Antibodies can likewise repress tumour development most effectively when they bind to oncogenic growth factor receptors (e.g., HER2/NEU and EGFR) and draw in macrophage or NK cell-actuating Fc receptors or enact the complement proteins' cascade [9].

The theory of “seed and soil” given by Stephen Paget was considered the first research, as back as in 1889, on the tumour microenvironment (TME). As per the theory, seeds (tumour cells) were grown on different soil (organ), that is, now known as microenvironment (ME) [23]. It became the foundation for developing antiangiogenic and immunological therapies, targeting TME. It, along with its stromal components, plays a pivotal role in tumour progression, recent studies reveal. Hence, it is necessary to comprehend the TME which, in turn, will help in the discovery of novice targets for immunological therapies, vital for NSCLC patients [24]. As it is, their survival rate with predictable chemotherapy is rather low due to late diagnosis when the patients are in advanced stages. Intratumoural stromal cells and tumorigenic epithelial cells which are modified genetically have natural interactions as a result of heterogeneity of TME [25]. The primary attributes of cancer which include inflammation, angiogenesis, immune suppression, extracellular matrix (ECM), and metastasis are controlled by these cells [26]. Cancer and noncancerous cells of the TME interact via gap junctions, effector molecules, and tunnelling nanotubes [27].

**Figure 1:**
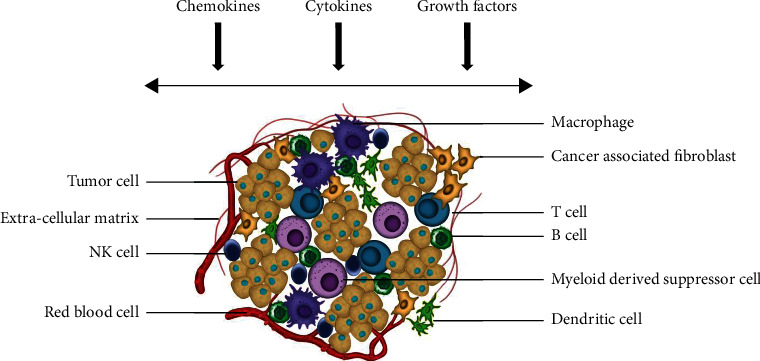
Tumour microenvironment and its components. The TME entails cellular as well as noncellular components. The former comprises endothelial cells (ECs), mesenchymal stem cells (MSCs), niche, immune cells, cancerous and noncancerous cells, cancer-associated fibroblasts (CAFs) and adipocytes (APs) which help in the growth of the tumour. The noncellular component consists of mediators like cytokines, chemokines, and growth factors. They can promote the progression of a cancer cell or get promoted by themselves.

In metastasis, cancer cells from their initial site of occurrence migrate to different locations, making it their abode for further growth. The tumour and the ME surrounding it begin a sequence of steps that make inroads, secure, and reproduce in a distant tissue, inducing metastasis. For a normal cell to turn into a tumour is context-based, meaning thereby a particular TME might induce tumour development while another may not. The TME encompasses cellular and noncellular components (Figure 1). Additionally, TME also includes ECM [26, 28, 29]. Collagen is the most abundant component of lung tissue ECM which provides tensile strength. When NSCLC modifies its appearance, the collagen structure, too, diverges contributing to the formation of a more appropriate ECM composition for the growth of the tumour. In NSCLC, cross-linking of collagen can likely be altered. An enzyme responsible for collagen cross-linking, lysizide-oxidase (LOX), in hypoxic conditions increases its presence. Invasion can be induced by the binding of LOX-associated collagen in NSCLC [26].

Lung cancer's proliferation, attack, and metastasis are intricate. The tissues of the tumour have innated genetic eccentricities which play a role in the spread and exchange with local ME's constituent immune cells. TME's immune systems, namely, innate and adaptive, work differently [30, 31]. The former, acting as the chief response system against the foreign invaders, constitutes macrophages (CD68+), NK-T-cells (CD56+ and CD3+), dendritic cells (DCs) (CD1c(+)), natural killer (NK) cells (CD49a, CD69, and CD103), and neutrophilic phagocytes [32]. Tumour metastasis, invasion, and angiogenesis are supported by cancer cells which promote tumour improvement in case of reprogramming of the system. However, the second, involving B-cells (CD20+) and two T-cells groups—Helper T-cells (CD4+) and Cytotoxic T-cells (CD8+), prompts the reduction of tumour progression [33]. The lung tumour niche is made up of several constituents—67% of TME is composed of tumour-infiltrating leukocytes (TILs), aiding in antitumour response. Tumour-associated macrophages (TAMs) are the next major constituents while pervading DCs and NK cells are in small numbers [34].

Another type of TILs, neutrophils which can infiltrate tumours, can display positive and negative tumoural effects. But in NSCLC patients, increased density of neutrophil can be indicative of bad prognosis. In the TME, the most abundant immune system linked stromal cell type is TAMs [5]. Since they are activated off and on, they lead to cancer progression, epithelial to mesenchymal transition (EMT), intrusion, and metastasis, resulting in shoddy prognosis. Malignancy in the TME is furthered by stimulation of macrophages and other stromal cells (non-malignant), like vascular endothelial cells and fibroblasts by cancer cells on being signalled [35]. By influencing treatment responses, cancer-associated fibroblasts (CAFs) streamline tumour growth, develop, and invade lung cancer. In the TME, CAFs induce angiogenesis, tumour propagation, infiltration, and metastasis by separating various growth factors like cytokines as a result of inflammatory cells' function inhibition [29]. Metastasis is promoted by TAMs' positive response. Malignancy is encouraged by TAMs but its explicit mechanism is not yet identified. This might be the reason for the usage of microfluidic chips in cancer studies due to its biological compatibility and inexpensiveness [36]. Moreover, it is easy to regulate cell growth and stimuli spatiotemporally, indicating its potentiality to be used as a TME mimicking model [37].

The prerequisites for metastasis include ME regulating molecules secretion, cell motility, and the start of EMT stimulating invasion in the exterior of the basement membrane; vessels of the lymphatic system and remote blood where intravasation occurs; circulating tumour cells (CTCs), essential for survival and migration to the lymphatics; secondary tissue where arrival and extravasation occurs; and secondary site occupation [28, 38].

The recognition of tumour cells at different cell niche is not an inherent scheme. Metastasis has multiple dimensions with its genetic and epigenetic modification effects exerted on the tumour cells and growth factors, new surrounding ME and relationship with other tumour cells [39]. A vital regulator of metastasis that EMT is, it constitutes a series of behavioural changes and phenotypic variations. This causes malignancy to develop from initial tumours. Also, anticancer agents' resistance developed by NSCLC can be attributed to EMT. Consequently, the solution for this lies in discovering novel molecular markers associated with NSCLC metastasis and EMT prognosis [26]. Invasion of cancer cell, its migration, and metastasis begin by a properly documented mechanism of EMT in which the phenotype anterior–posterior-polarised motile mesenchymal is acquired by epithelial cancer cells which are apicobasal-polarised. During EMT, there is a downregulated expression of an epithelial marker, E-cadherin, and diametrically opposite expression of the smooth muscle actin (a-SMA), a mesenchymal marker [40].

Of the 33% diagnosed NSCLC patients, EMT and exodus cause them to reach metastasis. Tumour stroma has a crucial constituent in the form of immune cells, facilitating cancer progression as they inhibit or promote metastasis and tumour EMT [41]. Immune cells like mast cells (MCs), macrophages, and lymphocytes provide conducive conditions for the growth of NSCLC. MCs having a part to play in relocation and tumour EMT are a fact [42]. MCs have always subsisted in the heterogeneous immune cells called bone marrow (BM). It acts through secreting preformed or newly-synthesised soluble modulators [43]. Inflammation supplies the tumour ME with bioactive molecules, stimulating chemokines, EMT, and growth factors [44]. They prevent cell death leading to cell proliferation and enzymes that modify ECM, angiogenic promoting factors that enable tumour angiogenesis, invasion, and metastasis, aggravating progression of cancer [16, 41].

It is said that roughly one million cancer cells per 1 g of tumour circulate inside cancer patients every day. Of which, just a fraction of them endures and reaches a distant location, forming a niche. So, encoding, programming, and adaptations intrinsically are required with key variations to aid the process of metastasis, indicating a substantial evolutionary hurdle that tumours need to surmount and control evolutionary obstacles in tumour cells [28].

The CTCs are invited by compelling ME using a premetastatic site to aid as an organotropism chaperon. Metastatic deposits and principal tumours release these cells into the bloodstream. CTCs, immune cells, and platelets along with macromolecules and small molecules form a functional unit called circulome [38]. Blood flow-and-NK cells-induced stress, due to the arrival of these cells in the bloodstream, can cause severe damage to the cells. Hence, the unfolding of the metastatic cascade by CTCs is challenging. CTCs can be protected by platelet-rich thrombin covered by tumour cell tissue-mediated platelet activation and coagulation cascade initiation [45]. Based on stimuli and TME, protein synthesis, exosome release, miRNA splicing, and membrane inflammation are the different types of platelet changes that can occur. CTCs' survival rallied by a platelet transcriptome is a signal of latent modification caused by capturing mutant RNAs from tumour cells or mingling miRNAs from TME by platelets. Such aggregates in which CTCs get entrapped are protected from immune surveillance by the CTCs. They, surrounded by these platelet accumulations, are hypothesised to have the following two situations: either tumour cells are completely submerged by platelets or in the midcluster amasses of homotypic origin are formed by platelets whose periphery is surrounded by CTCs [28]. Despite histological and cell markers persisting in metastatic cells, antineoplastic therapies are less effective which can be attributed to metastatic ME being dissimilar. Even after extravasation, blood vessels remain in contact with cancer cells, up to the point when the vasculature undertakes remodelling and cooption happens or when Vascular Endothelial Growth Factor-A (VEGF-A) tempts angiogenesis [28].

Tumour cells have a specific immune regulating method to safeguard themselves from the immune system, which allows them to evade T-cells carrying antigens, leading to tumorigenesis. T-cells are given to masking many proteins' expressions—T-cell immunoglobulin and mucin link to cytotoxic T-lymphocyte domain-3 (TIM-3) [46]. They also exhibit the same function over Cytotoxic T-lymphocyte-associated protein-4 (CTLA4) and Programmed Death Protein-1 (PD-1) along with inhibitory elements. At low levels in NSCLC, tumours positive for the death ligands are 20-60%. Ligand expression is visible on the plasma membrane of tumours and/or the protoplasm. Damage to T-cell function causes high levels of PD-1 [26].

At the time of tumour progression, one trademark is immune evasion, suggesting the involvement of immune effectors in the TME. In the absence of early detection, the maximum number of patients is left to face highly toxic treatments with little clinical advantage. The only option to arrest this problem is to first acquire efficient therapies while further studying the causes that dominate tumour growth and spread [22]. Attempts to restrain exchanges between the immune system and tumours have yielded worthy cancer therapies, like “Checkpoint blockade.” They impede negative regulators of T-lymphocyte role, enabling active responses against tumours. The trials at a clinical level, too, have discovered dramatic, lasting tumour regression among almost all cancers, lung adenocarcinoma (LUAD) included, necessitating many approvals [34].

When there is a healing wound or infection, inflammatory cells trigger a physiological response in the form of inflammation. Chronic swelling can cause persisting inflammation which can then lead to severe damage to the tissue due to cellular proliferation and cause dysplasia and metaplasia [47]. This is indicative of an important relationship between chronic inflammation and infection during initial neoplastic growth. This elevates the chances of malignant ME with inflammatory cells and growth factors more than the chances of malignant proliferation. Endothelium and fibroblasts (TILs), apart from local tissue cells, form the tumour ME. A research group involving clinical and epidemiological analyses indicated that one-fifth of the tumour cells was associated with chronic illnesses [33].

The immune system, to overcome surveillance, can pick and choose the cancer cells. Macrophages linked to cancer such as monocytes, NK cells, MCs, and innate immune system preserve carcinogenesis as the tumour necrosis factor (TNF) and proinflammatory ME mediated by interleukins (ILs), for EMT to take place, stimulate the Nuclear Factor- (NF-) *κ*B, helping other transcriptional factors [47]. Inside the tumour, neovascularisation and metastasis are promoted by mesenchymal (m-cars) looking and EMT experienced cancer cells. Nutritional components are provided by angiogenesis serving as a metastatic corridor. In angiogenesis, an important part is played by VEGF and VEGF receptor 2- (VEGFR2-) mediated signalling. For transpiration, EMT necessarily requires oxygen. If the tumour has regions of low oxygen then it can result in the expression of Hypoxia-Induced Factor (HIF) [48, 49].

Along with tumour carcinogenesis and outcomes, the development of tumour-associated inflammatory ME occurs, having two primary units: inflammatory mediators like chemokines and cytokines and different cells like an epithelial, macrophage, lymph, and tumour cells. There exists an established connection between illnesses like pulmonary fibrosis and chronic obstructive pulmonary disease with lung cancer [50]. Hence, molecular and cellular exchanges present in the inflammatory ME of NSCLC need to be extensively studied. This can help in preventing cancer along with checking metastatic processes and intrusions by hitting specific targets (Table 1) [51].

## 2. Cytokines/Chemokines' Roles in Tumour Progression and Immunotherapy

With lung cancer getting the better of all available treatment strategies, it is pertinent to look for some alternatives, especially when defence against cancer cells revolves around the immune system. TILs (immune cells) of the TME, particularly lymphocytes and macrophages, produce low-molecular-weight and nonstructural proteins called cytokines (<30 kDa) and chemokines which regulate many cellular activities. Such processes include metabolism, proliferation, cell and tissue repair, and chemotaxis that involve interaction with specific cell receptors which possess intercellular signalling through autocrine, paracrine, and endocrine modes of action [52, 53]. However, cytokines and chemokines are known to play significant roles in producing local as well as systemic inflammation. In the TME, these factors matter a lot in tumour progression and spread, besides therapy resistance. Both cytokines (IL-6, 10, 17, 27, 35; TNF-*α*; IFN-*γ*; TGF-*β*) and chemokines (CCL-2, 5, 18; CCR-4; CXCR-4; CX3CL-1; CXCL-1, 5, 8, 13) are very commonly targeted for lung cancer treatment, considering their biomarker roles [5].

IL-6, a cytokine, is secreted majorly by macrophages among other stromal cell types in the TME. Its hormonal action (autocrine and paracrine) is illuminated once it binds to IL-6R (ligand-binding receptor). Its function in the TME involves the mediation of several responses. It aids proliferation, apoptosis, invasion, angiogenesis, EMT, and metastasis via immunosuppression. It also relocates tumour cells to other new sites as wells as lymph nodes, rich in T-lymphocytes, according to a recent study [54]. T-cells are capable enough to move tumour from one state to another, say from suppressive to responsive and prevent progression and spread. TAM-based IL-6 provokes not just immune cell penetration but also cancer stem cells (CSC) growth and sustenance. In A549, a lung cancer cell line, IL-6 is known to use phosphatidylinositol-4,5-Bisphosphate3-Kinase/Threonine Kinase-1/AKT Serine (PI3K/AKT) signal to trigger its growth. At different stages of lung cancer, IL-6's mechanisms upregulated CCL-2/5 and played a part in the EMT and therapy resistance, a recent study in *in vivo* mouse and *in vitro* human lung tumour representations indicated [5]. Blockade of IL-6 modifies the TME, posing a hurdle in hitherto untried lung tumorigenesis models. Several methods have been used aiming IL-6 signalling routes—hindering antibodies and peptides against Signal Transducer and Activator of Transcription-3 (STAT3) stimulations, Janus kinase (JAK) phosphorylation, and IL-6, IL-6R, IL-6–sIL-6R complex [55]. In NSCLC, CAFs play an important role as they increase EMT signalling to arbitrate chemoresistance [26]. IL-6 production is known to be suppressed by IL-10 action [30].

Because of its capability to thrive and react to pathogens in a provocative atmosphere leading to necrosis and cytotoxicity in tumours, TNF-*α* has acquired the sobriquet of pyrogenic cytokine. It binds with its target cell using either of the two receptor families—TNFR1 and TNFR2 [5]. According to *in vivo* and *in vitro* lung cancer model studies, TNF-*α* triggers similar processes as IL-6. These include apoptosis resistance, proliferation, angiogenesis, invasion, and metastasis. The downregulation of Cyclin-dependent kinase inhibitor 1A (CDKN1A) induces doxorubicin treatment producing TNF-*α*. This, in turn, prompts TP53-deficient lung tumour apoptosis. TNF-*α* in the TME persuades the crosstalk between TAMs and other cellular components which controls survival and growth routes besides triggering programmed cell death through TNFR1 [56]. Despite TNF-*α*'s significance of being antitumorigenic—it reduced the tumour growth—its side effects were the aberration. So much so, several pieces of research cautioned against manipulating TNF-*α* when other studies pitched in on upgraded worthiness [57].

The discovery of T helper 17 (Th17) cells as a third subset of T helper cells in 2005 altered the traditional Th1/Th2 T helper cell differentiation paradigm. Th17 cells are distinguished from other T-cell lineages by their development of IL-17, expression of specific transcription factors, and performance of specific biological functions. The unique cytokine combination of TGF- and IL-6 is needed for mouse Th17 cell differentiation. Furthermore, IL-6 stimulates the synthesis of IL-21, which works in concert with TGF and IL-23 to promote Th17 cell differentiation in mice. The early differentiation of murine Th17 cells requires and is aided by IL-1. Human Th17 cell differentiation requires IL-1, and the ideal cytokine milieu for human Th17 generation is a combination of IL-1, IL-6, and IL-23. In addition to cytokine regulation, molecular programming of transcription regulation is a determinant of Th17 growth. Signal transducer and activator of transcription 3 (Stat3), retinoid-related orphan receptor gt (ROR-gt), nuclear receptor ROR-a, IFN regulatory factor 4 (IRF-4), B-cell-activating transcription factor (B-ATF), and hypoxia-inducible factor 1 are all essential for Th17 cell growth (HIF1-a) [58].

A growing body of evidence indicates a link between chronic infection and inflammation and tumorigenesis. Local inflammation in the tumour microenvironment attracts a variety of immune cells, including ab T cells, gd T cells, and natural killer (NK) T cells, which can all play important roles in tumour immunity. The presence of Th17 cells in a tumour microenvironment is predicted, given that Th17 cells have been recognised as important players in the immunopathogenesis of inflammation. Despite the fact that Th17 cells are abundant in tumour microenvironments, their function in tumour immunity is debatable. The majority of studies looking into the connection between Th17 cells and cancer have used mouse models, with mixed results [58].

IL-35, another immunosuppressive cytokine, belongs to the IL-12 family. It is the only member that is expressed majorly by Regulatory T-cells (T_regs_). IL-35 mediates signalling through STAT1 and STAT4. It does so by binding to its receptor IL-35R. IL-35 suppresses T-cell proliferation and effector function. Many reports indicated the T_regs_ induced by IL-35 in the TME constraints NK cell's antitumour activity and functions of CD4 and CD8 [59]. It upregulated the production of IL-10 and TGF-*β* to do so. Concurrent to these findings, it was also reported that increased levels of IL-35 in the plasma were linked to disease progression in lung cancer patients. The blockade of immunosuppression by IL-35 using specific monoclonal antibodies could be considered a potential therapeutic target [60].

IL-10, a cytokine with anti-inflammatory action, is secreted by stimulated B and T-lymphocytes and macrophages. It acts by binding to its receptor IL-10R [55]. Its major role is to restrain the classical macrophage activation. It suppresses the generation of IL-1*β*, IL-6, IL-8, IL-12, TNF-*α*—proinflammatory cytokines—and Granulocyte-macrophage Colony-stimulating Factor (GM-CSF) [30]. Besides, in activated macrophages, it reduces the expression of major histocompatibility complex-II (MHC-II) by inhibiting antigen presentation. Additionally, IL-10 inhibits the production of IFN-*γ* (Interferon-*γ*) by NK cells and T_h_1. In various cancers, serum engagement of IL-10 was very much evident, concurrently in immune-stimulation and immunosuppression. TAM- (M2-) based IL-10 presented constant prognostic prominence in lung cancer patients. Though there are some similarities between IL-10 and TAM-based IL-10, both having been experimented with, the former's signalling imprints tread intricate molecular system consisting of 76 reactions and 37 molecules (minimum) to back cancer expansion [5, 61]. TAM-based IL-10 tends to endorse the stemness of lung cancer. In *in vivo* tumorigenesis mouse model studies, it used NFKB/JAK1/STAT1/NOTCH1 signalling pathways. It also aids *in vivo* lung cancer progression and metastasis. It targets upregulation of CCL-2/CCR-2 and CXC3CL-1/CX3CR-1 axis in macrophage-tumour cell crosstalk [36]. This signalling barrier of IL-10 is not only under serious probe but several strategies, too, are going through clinical appraisal. Though IL-10 is a well-studied cytokine, the role it plays in cancer therapy is still unelucidated [62]. Different approaches like developing monoclonal antibodies and blocking peptides, against IL-10, are undergoing various pathological screening. Some other plans for receptor-blocking (IL-10R) and small-molecule inhibitors which aim at the signalling of JAK/STAT3 are also being undertaken [63]. Some evidence pointed to the inhibitory role of IL-10+ B_regs_ (Regulatory B-cells) in human lung cancer, especially in ADC. They are known to be linked to tumour progression [33]. Besides, IL-10 is also considered very important to maintain anti-inflammatory regulatory T-cells (T_regs_) homeostasis. It also plays a vital part in the clampdown of IL-17-expressing T-cells (T_h_17), proinflammatory in nature [64]. The sense of balance maintained by various T-cell populations between pro- and anti-inflammatory signals is crucial for the suppression of tumour progression [64]. Despite various roles, IL-10's capability in downregulating MHC I leads the tumour cells, NK-sensitive [30]. This was indicative of IL-10 with a combined effect of stimulatory cytokines like IL-2 and might trigger NK cell-mediated immunity against tumour cells. Yet conflicting evidence on the part of IL-10 in cancer is emerging. One plausible reason, as cited by Konjević et al., explains that its function is maintained by not just environmental factors but a balance of cytokines too [65].

Myeloid-derived suppressor cells (MDSCs) play an inhibitory role leading to antitumour immune response, promoting an immunosuppressive tumour environment [66]. Since the 1990s, after the discovery of IL-10, its role in cancer immunotherapy has raised eyebrows thanks to its complexity. In many cancer types, it is known to aid in tumour progression (HIV-positive cervical cancer [67]), while in gastric adenocarcinoma [68] and prostate cancer [69] cases, diminished levels of IL-10 are found to be a high-risk factor. A study by Lee and colleagues suggested that IL-10 inhibits the IL-6/STAT3 axis on MDSCs and weaken tumour progression [62]. IL-10 is also considered to employ stimulatory as well as inhibitory effects on T-lymphocytes. A study by Fujii et al. [70] suggested that an administration of IL-10 right after a booster vaccine heightened antitumour immunity and improved vaccine efficacy. Their adoptive transfer studies using spleen cells indicated that IL-10's ability to maintain the function of CTL could be improved by lessening CD+ T-lymphocytes. This result pointed to the opposing effects of IL-10 on CD4+ and CD8+ T-lymphocytes leading to immune-suppression and immune-manifestation, respectively. This dual part played by IL-10 suggests its role in coming up with improved and advanced anti-cancer vaccines [70]. Many studies have indicated the development of deficient signalling of IL-10 in patients with lymphomas at a young age while some other studies revealed the development of colon cancers in IL-10 mice knockout experiments [71, 72]. Such researches indicate the repercussions of IL-10 deficiency to understand tumour-promoting inflammation and IL-10's crucial role in inflammation control [64].

Another anti-inflammatory cytokine, IL-27, is known to act as a tumour suppressor. This immune-enhancing two-chain cytokine propels T_h_1 cell differentiation, CD4 + T proliferation, and IFN-*γ* production coherently with IL-12. It possesses a pleiotropic effect comprising IL-27p28 and EB13 subunits. IL-27 uses STAT1 and 3 majorly. It comprises IL-27R*α* and gp130 chains. In NSCLC, it restrains the growth while increasing apoptosis. In xenotransplant models, IL-27, besides downregulating stemness-and-EMT-linked genes, does force intratumour myeloid cells to pressure antitumour effects [73]. The combination of IL-27 and Apricoxib, a COX2 inhibitor, impedes EMT in NSCLC cells, working in a STAT1-dependent manner. A novel research pointed to IL-27 stifled NSCLC cell growth, relocation, and intrusion. Yet another anti-inflammatory cytokine, IL-37, a member of the IL-1 family, constitutes 5 splice variants—IL-37a to IL-37e. The second type, IL-37b, is the most widely researched isoform as it is expressed extensively in many human organs and tumour cells [74]. It has been indicated as an inhibitor of both the innate immune system and inflammation. It is known to suppress proinflammatory factors like IL-1*α*/*β*, IL-6, and TNF-*α*. IL-6/STAT3 pathway suppression, *β*-catenin suppression, pSmad3C/P21 tumour-signalling suppression, and CD57+ NK recruitment are said to be the underlying mechanisms for the action of IL-37 [75].

Tumour growth factor (TGF-*β*), another anti-inflammatory cytokine aiding in angiogenesis and metastasis, is secreted by M2 macrophages. It uses types I and II transmembrane serine/threonine kinase receptors to modulate its biological functions. Tumour growth promoted by Cyclooxygenase Prostaglandin E2 (COX-PGE2) and VEGF mediators are produced by these macrophages. There is another subgroup of macrophages, M1, that secrete IFN-*γ*, an inflammation-promoting cytokine, reactive nitrogen, and oxygen intermediates, inducible nitric oxide synthase expression and MHC molecules indicating a correlation with NSCLC [52]. Its signalling pathway possesses pleiotropic roles. Such roles modulate not just the immune response but also cell growth, differentiation, apoptosis, motility, and invasion. TGF-*β* aids in differentiation and immunosuppressive cells induction and subsequent inhibition of T and anergic NK cells functions in TME. These immunosuppressive cells are also the main source of TGF-*β*. As TGF-*β* remodels TME that promotes tumour progression and metastasis, it could act as a potent therapeutic agent to enhance antitumour immunity mediated by NK cells. TGF-*β* utilises JAK/STAT3 signalling pathway to induce EMT in lung cancers [76].

The dysregulation of another cytokine, IL-17, is connected to many human diseases like inflammation and cancer. It is a six-member cytokine family—IL-17A to IL-17F—with diverse order homology and functions in host immune responses. IL-17 receptors (IL-17R, IL-17RA to IL-17RE) are bound by these cytokines, operating as homo- or heterodimeric complexes. T_h_17 cells produce IL-17A, a first member of the IL-17 family [63]. Its overproduction displays a significant association with not only autoimmune and chronic inflammation disorders but also cancer [77]. A signalling cascade, IL-17B/IL-17RB pathway encourages not just cancer survival but also aids in its proliferation and relocation. The IL-17B/IL-17RB pathway's protumour functions are different and intricate, owing to the processes that perform unswervingly on tumours apart from unintended mechanisms which force TME reshaping [63]. IL-17B signalling is crucial in aiding tumour survival and growth, many *in vitro* and *in vivo* assays identified. Every study point to the hindrance of the IL-17B/IL-17RB axis through downregulation of receptor expression in tumour cells, utilising diffusing anti-IL-17RB antibodies, reestablished *in vitro* and *in vivo* chemosensitivity [77]. Remarkably, IL-17RB providing signal across ERK/GSK-3*β*/*β*-catenin route is connected to lung cancer's EMT [78].

Both mouse tumour models and human cancer patients have shown protumour activity mediated by IL-17 and Th17. Angiogenesis and cytokine activation in the tumour microenvironment are the key pathways responsible for IL-17 or Th17 cells' protumour activity, resulting in tumour growth promotion. In nude mice, IL-17 was found to promote tumorigenicity of human cervical tumours, which was linked to increased levels of IL-6 and IL-8 and macrophage recruitment at the tumour site. Furthermore, a study in a mouse model of colon adenocarcinoma found that IL-17's antitumour activity was linked to its ability to induce tumour angiogenesis by inducing a wide variety of angiogenic factors from fibroblasts and tumour cells, including VEGF, PGE2, keratinocyte-derived chemokine, and nitric oxide. In vivo, IL-17 increased net angiogenic activity and the growth of human nonsmall cell lung cancer by encouraging CXCR2-dependent angiogenesis, according to the same research community. In addition to its role in angiogenesis, IL-17 can trigger the development of IL-6, which triggers the oncogenic signal Stat3, leading to an increase in the expression of prosurvival and proangiogenic genes [58].

### 2.1. Key Findings Supporting the Role of Cytokines in NSCLC

IL-6 is crucial for the growth of NSCLC, according to a recent study in its progression. T-lymphocyte IG domain and mucin domain 4 (TIM-4) played a key part, notwithstanding the contribution in NSCLC migration promoted by IL-6 by TIM-4. It was observed in NSCLC tissues that for the prognosis of TIM-4 manifestation, IL-6 could be an autonomous prospect. All these conclusively proved that TIM-4 engrossed in IL-6 supported relocation, foray, and EMT of NSCLC [79]. In other words, TIM-4 is an essential molecule in IL-6 overexpressed cancers and enhancing metastasis, therefore, pursuing it could turn out to be an effective pharmacological goal in IL-6 excessively pronounced cancers. In short, the studies without a doubt focused on the impending function of TIM-4 in augmenting metastasis of NSCLC [80]. IL-6, a part of numerous biological factors, is released along with VEGF and Matrix Metalloproteinases (MMPs) by Toll-like Receptors (TLRs) when tumour cells dodge immune surveillance [81]. The notable difference between normal and lung cancer tissues was that these factors were a part of the Differentially Expressed Genes (DEGs) [82]. Hence, a recent study stressed on therapeutic modalities by targeting lung tumour TLR signalling pathways [1]. A few other studies also suggest targeting the TGF-*β*-IL-6 axis to arbitrate the resistance mechanism of cancer cells for better molecular therapy [83].

An important study by Lee et al. [62] confirmed another prospective anticancer biomarker, that is, inhibition of IL-6/STAT3 axis. They observed a spurge of IL-6 along with high tumour progression in comparison to the wild-type mice when injected with TC-1 (cervical cancer) cell line. They even observed a high level of anti-IL-6R mAb and MDSCs (they inhibit tumour progression) in IL-10-deficient mice. They concluded with the result that indicated an effective treatment target with a combination of anti-IL-6R and STAT3 inhibitor as this was shown to reduce the tumour progression further [62]. The serum level of IL-10 seemed to be a predictive marker in NSCLC at a later stage, said De vita et al. [84] and Soria et al. [65], reported in NSCLC patients, having a low level of IL-10 expression at the early-stage, considered a shoddy projection. The findings of a recent study signified the role of the IL-10/JAK1 signal pathway as an effective target for NSCLC treatment. The study pointed to the promotion of CSC-like properties of NSCLC cells by TAM-based IL-10 [45]. IL-10 does this by triggering the NF-*κ*B/JAK1/STAT1//Notch1 signalling. The effect of such pathways can be choked by constraining these signalling molecules. The reported signalling molecules, when expressed at high levels, are connected to poor patient predictions [65]. In one study, the role of IL-27, another anti-inflammatory tumour suppressor, was elucidated. The functional relationship between IL-27 and miR-935 showed that upregulation of the latter amounted to promoting the survival ability of and metastasis of NSCLC cells by targeting the expression of IL-27. The data in this study suggested that by targeting IL-27 in NSCLC, mi-RNA-935 acted as an onco-miRNA. Hence, miR-935 inhibition is a likely therapeutic target [85]. Jiang et al., in their study, elucidated the role of IL-37 being downregulated in NSCLC patients' serum. They also saw a negative correlation with TNM (tumour, node metastasis) stage and said that IL-37 might be able to suppress the invasion and metastasis of NSCLC. They also suggested that IL-37 could partially inhibit the STAT3 activation by diminishing EMT expression through IL-6 inhibition. Hence, they concluded that IL-37 might be a potent and novel tumour suppressor in patients with NSCLC [86].

A recent study indicated that in *in vivo* models, IL-17B conveyed directory through its IL-17RB and encouraged cancer cell survival to proliferation to relocation to resisting usual chemotherapeutic agents. This study also identified the alteration of TME by IL-17B signalling and subsequent tumour growth induction. Besides, this finding linked the role of IL-17B with poor prognosis in lung cancer patients among many other types, identifying clinical relevance [77]. Despite the potential role of the IL-17B/ILRB trial in lung cancer, microarray dataset analysis in a study was linked to IL-17B and IL-17RB gene expression indicating least patient survival. Moreover, IHC analysis of IL-17RB is upregulated in patients with lung ADC and is related to lymph node and metastasis—reduced progression-free and complete survival [56]. A study by Yang et al. [78] successfully established that how IL-17RB-intervened initiation of the ERK pathway in lung cancer cell lines was vital to sustaining the expression of two important transcription factors for EMT orientation Twist and Snail. Upon IL-17RB knockdown in the lung cancer cell lines—A549 and CL1-5, the expression of Twist and Snail declined. Stimulation of the ERK/GSK-3*β*/*β*-catenin path after IL-17RB encouragement did promote the intrusion and relocation of human lung cancer cell lines—H441 and CL1-0, in *in vitro* studies [87]. Further, IL-17RB's excessive expression in the H441 cell line majorly increased the total lung metastatic nodules of mice (xenografted). Hence, in lung cancer, the level of expression of IL-17RB correlated significantly with distant metastasis and lymph nodes [88]. A study to determine the protumour effects of IL-17 or Th17 in human nonsmall cell lung cancer (NSCLC) when subcutaneously injected into SCID mice showed transfection of IL-17 increased NSCLC growth in vivo (SCID mice) via promoting CXCR2-dependent angiogenesis. Another study showed the antitumour effect of IL-17 or Th-17 in human lung cancer when human tumour antigen MAGE-A3-specific Th17 cells converted into *in vivo*. IFN-g secreting cells as they differentiated into effector T cells. In yet another study, the accumulation of Th17 cells in human malignant pleural effusion predicted improved patient survival [58]. From the Leucine-rich repeat (LGI) family of proteins, LGI-3, a proinflammatory cytokine is prevalent in lung and many other cancers. The role of LGI-3 in NSCLC, according to a study, pointed to the dysregulation of LGI-3. Its consternation in the cell-cell communication in the ME and cytokine network was even more visible. In NSCLC, the deregulated expression of LG13 also meant the rise of appearance in seven of the genes, namely, NCF1, NCF2, CSF3, IL-6, CCL-3, PTGS2, and CXCL-2. LGI-3 was no different when it came to increasing M1-polarised macrophage markers, aiding in antitumour processes in the NSCLC ME (Table 2) [89].

Soluble TNF-*α* receptors from the blood can be removed to boost endogenous TNF-*α* activity, and this is done using a single chain TNF-*α*-based affinity column developed by Immunicon Inc. In the experimental cancer model, the efficacy of the agents was improved by a low dose of TNF-*α* pretreatment. This treatment preceded the use of cisplatin, gemcitabine, and paclitaxel chemotherapeutics. Yet, a few other studies proved penetration of TAMs along with TNF-*α* (cytotoxic M1 phenotype) in islets of tumours benefited in NSCLC and other malignancies [57]. In NSCLC phase II trial, a TGF-*β*2 antisense DNA allogeneic tumour cell vaccine has been elucidated. It showed promising clinical gain [30, 90].

## 3. Role of Chemokines in Lung Cancer Therapy

They, area family of chemoattractant cytokines, play their role in immune cell exodus and modify the immune microenvironment. These small proteins bind to and activate G-protein Coupled Receptors (GPCRs) [91]. It has an extended family of *α*-chemokine (CC), *β*-chemokine (CXC), *γ*-chemokine (C), and *δ*-chemokine (CX3C) [92]. Chemokines are a family of minuscule, secretory, and fundamentally correlated cytokines playing a major part in immunity and inflammation [89]. Meant to determine the conformation of stroma of the tumour, these factors were seen explicitly affecting the growth and metastasis of cancer cells. Two families of chemokines—CC (CCL-2, 3, 5) and CXC (CXCL-1, 2, 5, 6, 8)—employ CCR-2 with monocytes and CXCR-2+ with neutrophils, at the tumour spot which distinguishes between TAMs and tumour-associated neutrophils (TANs), wielding either pro- or antitumour response [42, 93]. As stated above, some chemokines can modify leukocyte initiation—CXCL-16's action on CXCR-6, inducing macrophage polarisation giving rise to a protumoural role in solid tumour cells. CXCL-9 and CXCL-10 are strappingly linked to the immune response of T_h_1 via inducting CD4+ T_h_1, CD8+ Tc (Cytotoxic T) cells, and NK cells for antitumoural responses. Besides, there are DCs attracted by CCL-5, CCL-19, CCL-20, CCL-21, and CXCL-12 chemokines which employ regulatory T_regs_ and CCR-7+ DC.CCL-17 and CCL-22 working on CCR-4 are capable of straightaway recruiting T_h_2 and T_regs_ for tumour progression and spread [94–96].

Likewise, CC and CXC chemokines do have their roles to play in tumour progression and metastasis. CXC chemokines are classified into ELR- chemokines with angiostatic and ELR+ chemokines with angiogenic results. At the N-terminal of CXC chemokines with Glutamic-leucine-arginine (ELR) theme is the determinant of this classification. A few CC chemokines like CCL-2, 11, 16, 18 along with CXCL-18 push endothelial cell endurance and tumour angiogenesis [97]. CXCL-16 while dealing with CXCR-6 makes it an effective angiogenic moderator. CCL-2 and CXCL-12 can aid in angiogenesis and impede endothelial cells' apoptosis through straight receptor (CXCR-4 and CCR-2) binding on tumour vessels or obliquely propping up drafting in leukocytes. In contrast, chemokines like CCL-21and ELR− chemokines (CXCL-4, 9, 10, 11) come in the way of angiogenesis and endothelial cell growth. CAFs and penetrating leukocytes, obligated to chemokine receptors, are directly involved in cancer cell proliferation triggering PI3K/AKT/NF-*κ*B and MAPK/ERK pathways besides helping the survival of tumour cells by hijacking programmed cell death mechanism, striking coherence between apoptotic (pro- and anti-) molecules [52].

CXCLs/CXCR-2 axis is a vital chemotactic factor in cancer, known for the conscription of immunosuppressive myeloid cells from peripheral blood lesions or BM. Blockade of this axis showed improved prognosis in various disease models, including cancer [98]. Inhibition of CXCLs/CXCR-2 axis was recognised as a potent treatment target to either contain cancer cell growth and spread or improve ICB efficiency, according to many preclinical experiments. Hypoxia-induced CXCR-2 expression on tumour cells is time-dependent, contributing to the survival of tumour cells through NF-*κ*B and HIF-1 signalling. In a study, knockdown of CXCR-2 expression in cancer cells could improve the efficiency of paclitaxel, reducing metastases in the lungs. However, an elevated level of CXCR-2 is linked to diminished expressions of E-cadherin and *β*-catenin in cancer tissues, attributing to the part played by CXCR-2 in EMT [99].

Human Dachshund Homolog 1 (DACH1) was pronounced as a relevant tool against NSCLC while searching for lung tumour clampdown. It is a key constituent of the Retinal Determination Gene Network (RDGN) [17]. DACH1 is known to be involved in cell propagation, progression, and apoptotic mechanisms. DACH1 and mRNA protein expression levels are diminished in various tumour tissues as in lung cancer when compared to their levels in normal tissues. Genomic deletions and promoter region hypermethylation are the causes of the decreased DACH1 level. Apparatus investigation exposed that DACH1 contributed to not just the EMT and negative regulation of cell cycle but also played a major role in the reduction of CSC subpopulation. Many cytokines/chemokines such as IL-6 and CXCL-5/8 are secreted by the action of DACH1 [100].

CCL-2/MCP-1, belonging to the CC family of chemokines, is a powerful monocyte/macrophage chemotactic and one of the most studied chemokines relating to human diseases with the possibility of treatment regimes. It is either discharged in an organised way or through the introduction of oxidative stress and decipherable (soluble) factors. CCL-2 exerts its effect via paracrine or autocrine mode, binding to CCR-2 receptor [94]. The manifestation of an assemblage of IL-6 and IL-10 or CCR-2, CCL-2 connects to a shoddy projection in patients with lung cancer. In different cancers, crosstalk between TAMs and tumour cells play various roles in their growth through the CCL-2/CCR-2 axis [95]. They include tumour growth, EMT, penetration, and spread apart from monocyte/macrophage employment at the site of the tumour. CCL-18, another chemokine belonging to the family of secreted proteins, CC possesses inflammatory and immune-regulatory roles. In cancer, it is said to aid in tumour progression by modulating the TME. It is mainly secreted by monocytes, macrophages, and immature DCs [99].

CX3CL-1, despite being the only identified member from the CX3C chemokine family, it caught the imagination of researchers as it possessed dual functionalities of chemoattractant and binding molecule. Its role in propelling the relocation of T-cells, NK cells, monocytes, and MCs to the spot of action in a variety of clinical settings is vital.

CXCL-1, another chemokine, belonging to the CXC family, is operated by several signal paths and TME. TNF and VEGF are the major driving factors of CXCL-1 expression acting through PI-3K/AKT, JNK, and p38 MAPK signalling mechanisms in human lung carcinoma's epithelial cells. CXCL-1 uses the receptor CXCR-2 [5]. CXCL-1 interacts with CXCR-2 enhances proliferation in malignant carcinomas and chemoresistance [17]. The interface between tumour cells and neutrophils raised the manifestation of CXCR-4,7; MMP12,13; TGF-*β*, IL-6, the metastasis-related genes. With consistent results, circulating CXCL-1 in metastatic patients was rather high compared to the patients in the stage IA-IIB of NSCLC. On the other hand, hindering CXCR-2 suppressed angiogenesis, tumour growth, and metastasis and boosted chemotherapeutic response. CXCL-1 added to the tumour-linked neutrophils infiltration in lung cancer [17]. CD11b (+) Gr1(+) myeloid cells are pushed by CXCL-1/2 into the tumour, leading to the production of chemokines like S100A8/9 which augment chemoresistance, metastasis, and cancer cell survival. A new study, consistent with their previous results, confirmed elevated CXCL-1 levels in late-stage NSCLC patients than those of in IA-IIB stage NSCLC. One other finding was that CXCL-1 protein was found in higher amounts in ADC patients compared to other types of lung cancer. This rules that CXCL-1 could be used as a gauge to monitor cancer development. Specifically, uncovering the circulating CXCL-1 is an appropriate and satisfactory method [101]. CXCL-5, another chemokine, belonging to the proangiogenic subgroup of CXC family having an ELR motif comparable to that of IL-8, is also known as Epithelial-derived Neutrophil-activating Peptide-78 (ENA-78) [102, 103] and is found to play a vital function in leukocyte placement and tumour growth and metastasis. It functions by binding to its GPCR, CXCR-2. It aggravates cancer progression by enabling RSK1/2/AKT/ERK with EGFR pathways and stimulating the HSP27 (Heat Shock Protein) phosphorylation [104]. Another CXC chemokine, CXCL-13, secreted by follicular DCs and T_h_ cells is known to be responsible for the infiltration of B lymphocytes into the tumour, studies revealed. Research indicated the significant increase in B-cell-influx in lung cancer tissue in comparison to normal tissues. The correlation of tumour-infiltrating plasma cells and follicular B-cells with long-standing survival of lung cancer patients suggested the defensive actions of plasma cells in antitumour immunity antibodies [33].

Another important chemokine of the CXC family of chemokines is IL-8/CXCL-8 and is majorly secreted by macrophages and has a proinflammatory function. It exercises its influence by obligating to the heterotrimeric GPCRs—CXCR-1 and CXCR-2 [5]. Its receptors are articulated by a variety of cell types including monocytes, neutrophils, and ECs. Stromal and tumour cells in the TME are also known to express CXCRs. IL-8, having the authority for migration and instigation of all these cells, has its role cut out in the progress of lung cancer [84]. IL-8 is predominantly secreted by MCs, and high levels of MCs indicate a shoddy overall survival in NSCLC patients [43]. Yet, it can be a prospective biomarker to forecast tumour burden, apart from treatment response and survival of patients suffering from lung cancer [84]. In the lung TME, the countenance of IL-8 mRNA, persuaded by penetrating macrophages through the NF-*κ*B pathway, expressively compares with amplified angiogenesis and average subsistence of lung cancer [99]. CXCL-8 uses autocrine and paracrine secretions to fire major oncogenic signal in the TME (e.g., PI3K, RAS/Mitogen-activated Protein Kinase (MAPK), and JAK/STAT). This necessitates a precise probe into TAM-derived IL-8's molecular role in TAMs-tumour cell crosstalk. Blockade of IL-8-defusing antibodies (HuMax-IL-8 and ABX-IL-8) and its allied reactions along with small-molecule inhibitor of CX3CR-1/CX3CR-2 (Reparixin) will also aid in the investigation [105]. Tumour cell-TAM induced unwanted signals relay on factors like chemokines, cytokines, and others have an important part to play. In intratumoural NK cells, expressions of granzyme B and IFN-*γ* are restricted by a discharge of soluble factors by NSCLC cells which have been proved by studies. NK cells which infiltrate tumour have shown proangiogenic activities, producing VEGF and IL-8/CXCL-8 [84]. If the elevated occurrence of T_regs_ and lesser rate of NK cells were found in the malignant cases, the condition was vice-versa in nonmalignant areas, indicating robust cytolytic movement ex vivo. These specifics—adaptive and innate immunity—formulated NK cells, a striking object for therapeutic development [102] (Table 3).

### 3.1. Key Findings Supporting the Role of Chemokines in NSCLC

One study stressed the involvement of CCL-2/CCR-2 signalling in tumour progression and metastasis in lung cancer patients [104]. This topical study based its results not only on human lung cancer biopsies but also on coculture models and *in vitro* TAM-tumour cells. On the contrary, the intrusion of TAMs was heightened by NF*κ*B1-CCL-2 in the neddylation pathway in *in vivo* metastasis. The CCL-2 barrier lessened the progression by remodelling TAMs to M1-like phenotype and triggering CD8+ T cells. This was observed in an orthotopic and flank lung tumour model [104]. It has become a worthy immunotherapeutic ploy for use in many human illnesses. Hence, treatment regimens in TAMs to block CCL-2, CCR-2, and CCL-2/CCR-2 facilities are broadly studied. A new study by Bakouny et al. showed that tumour-derived microvesicles (TMVs) drawn from NSCLC cells were engulfed into MCs. It triggered the discharge of TNF-*α* and monocyte chemoattractant protein 1 (MCP-1)/CCL-2. Besides, it improved their activity of chemotactic/chemokinetic. Data also showed an upsurge of migratory cells. This occurred when the activated cells were pushed towards NSCLC-TMV, pointing to the chemotactic and chemokinetic functions of NSCLC-TMV [106].

MCs regulate anti- or protumorigenic roles by infiltrating many cancer cells. A recent study indicated that NSCLC cells drew its strength from MCs, releasing C-C family CCL-5 which mounted the CCR3 receptor on the MC surface [107]. While promoting tumour EMT and metastasis, unceasing inflammatory responses can also influence restrained cancer growth, involving CCL-5 in human NSCLC samples, said the study. It also pointed out that CCL-5 negotiated TGF-*β* appearance, connected with lung ADC forays [98]. Cancer-linked bone obliteration in NSCLC, the upstream receptor of CCL-5, Runt-related Transcription Factor-3 (RUNX3), was rather helpful when expressed a little. A few other studies proved that human NSCLC, exhibiting an MC infiltration, can have two extremes—worth complete disease-free and overall survival. The study concluded that a high concentration of MCs is positively linked to bad prognosis [43]. NK cells promoted immune control of tumours by upping the intensity of the orthodox type-1 dendritic cells (cDC1s) in tumours via two cytokines'—FLT3LG and CCL-5—production, as per recent studies [34].

In NSCLC tissues, another new study suggested the significant elevation of CCL-18 expression in comparison to nearby normal lung tissues. Another study by Plönes et al. [108] emphasised the elevated CCL-18 serum level in NSCLC patients. It also forewarned a decreased survival time in ADCs. Yet another study by Huang et al. [109] supported these observations in NSCLC patients when they saw an elevated CCL-18 serum level. The researchers concluded a positive correlation of increased CCL-18 level with the expression of Carcinoembryonic Antigen (CEA) and Cytokeratin 19 Fragment (CYFRA21-1). These are two of the lung cancer biomarkers. They pointed to a negative correlation to their survival time, as well, considering serum CCL-18 as a biomarker for the prognosis and diagnosis in patients with NSCLC [109].

Liu et al. [17] established in lung ADC patients that elevated CX3CL-1 mRNA visage was an optimistic prognostic pointer. Su et al. [17] concurred with the previous study confirming the presence of CXC3CL1 level in lung ADC but not in SCC. Similarly, in lung cancer, the CX3CL-1 expression was heightened. It also showed complicated clinical results with lymph nodes metastasised. The intrusiveness of human endometrial stromal cells (ESCs) is increased by CX3CL-1-induced M2 macrophage polarisation [17]. This is conducted by the upregulation of MMP9 and Tissue Inhibitor of Metalloproteinases- (TIMP-) 1, -2. Also, P38MAPK and integrin*β*1 signalling play similar roles. TAM-tumour cell crosstalk, particularly in *ex vivo*, *in vitro*, and *in vivo* replicas, was critical to the extent that it caused an explosion in lung tumour metastasis. This pointed to the urgent therapeutic intervention. Mutations transferrals like Epidermal Growth Factor Receptor (EGFR) and Anaplastic Lymphoma Kinase (ALK) are characteristically linked to poor patient prognosis and tumorigenesis [100].

Through interaction with CX3CR-1, CX3CL-1 intervenes its cellular effects. CX3CL-1 was prominent in lung cancer because of its increased presence along with SCM-1*β* as opposed to normal tissues, said a study by Zhou et al. [101, 110]. In lung ADC, expression of CX3CL-1 mRNA was evocative of an enhanced prediction, revealed another research. It directed majorly to the diminished levels of CX3CL-1 and CX3CR1 mRNA in normal tissues when compared with patients affected by LUAD [17]. CX3CL-1 is said to bind CX3CR1+ tumour cells, aimed at organs, trigger movement of cancer cells. An improved prediction became possible when CX3CL-1 expressed excessively in LUAD, leading to chemotactic efficiency and increased immune effector-cell penetration. More future research on LAUD patients may lead to prognostic worth of CX3CL-1 [111, 112].

A latest research showed a significant link of CXCL-1 in recurrent patients of NSCLC after undergoing surgery. CXCL-1 is known to induce T_reg_ cells' chemotaxis into the malignant pleural effusion, allowing the tumours to escape the immune response. Many studies contributed to the fact that abnormal regulation between T_h_1 and T_h_17 is key in NSCLC. This is particularly evident in the irregular IL-17 expression contributing to shoddy prognosis. Moreover, in NSCLC patients, the frequency of T_h_17 cells was interrelated positively to IL-6, IL-1*β*, and IL-23. Also, the frequency of T_reg_ cells frequency was also positively interrelated to TGF-*β*1 and IL-10 [17]. It was initially reported by Arenberg et al. [104] that elevated CXCL-5 level in NSCLC patients was associated with vascular density. According to one study, in NSCLC, upregulated CXCL-5 was linked to lymph node metastasis, increased expression of CXCR-2, very poor differentiation, and an advanced pathological stage. Additionally, it also indicated worse overall survival of patients where CXCL-5 was highly expressed [103]. Another study also supported with evidence indicating CXCL-5 expression was connected to cancer staging. In NSCLC models, CXCL-5 depletion was seen to inhibit tumour angiogenesis. It was also seen to attenuate tumour progression and metastasis [102].

Low-level of serum CXCL-8 is considered as a pointer of chemotherapy response for better estimates in different cancer types, including NSCLC stages II/IV. Its presence is also linked to tumour chemoresistance. CXCL-8 was seen to be stimulated by proinflammatory cytokines like IL-1 and TNF-*α*, barely detectable in healthy tissues [84]. Stimulated CXCL-8 can straightaway decrease the apoptosis of ECs while enhancing the proliferation of ECs. In the TME, the production of MMPs modulates angiogenesis to tear down the ECM resulting in the formation of blood vessels [102]. A few studies support the role of DACH1 as an antagonist of CXCL-8 in lung ADC. It suppresses tumorigenesis facilitated by CXCL-8. DACH1, in this case, aids in better prognosis [113]. IL-8 is known to reduce phosphorylation of *β*-Catenin degradation by inducing Wnt in tumour cells. This is known to trigger EMT leading to the relocation of tumour cells. In one study, the knockdown of *β*-Catenin led to a hampered Zinc Finger E-box-binding Homeobox-1 (ZEB1, EMT transcription factor) accumulation along with NSCLC cells relocation [102]. Cromoglycate, an MC inhibitor, might be a novice target to obstruct NSCLC cell migration. Another report pointed to the elevated production of IL-8 levels was stimulated by TAMs in NSCLC. It also suggested that IL-8 triggered angiogenesis in NSCLC cells. The impediment of this trail could be focused upon for therapeutic modalities in advanced NSCLC [43]. Even though a few *in vitro* studies stressed over the autocrine activity of IL-8, stimulating tumour progression, other studies pointed to the paracrine function of the tumour-derived IL-8 [105]. The latter was seen modifying the conformation of immune infiltrates in TME while inducing angiogenesis and tumour progression. Yet another study indicated the vital role of IL-8 in CSCs development [114].

## 4. Cytokines/Chemokines Exploit EVs for their Release

Extracellular vesicles (EVs) are membrane-enclosed sacs that are released from cells and contain biologically active cargo as well as cell type- and disease-specific molecular details. Furthermore, by manipulating cells in the local microenvironment, EVs have been shown to perpetuate disease pathogenesis and progression. There is a lot of interest in developing EV-based diagnostic and therapeutic platforms for cancer and other diseases because of their specific properties. However, before EVs can be fully utilised in medicine, a better understanding of the mechanisms that drive EV biogenesis is needed. We will review existing knowledge of EV biogenesis in cancer, address recent developments in the area, and provide evidence for the use of human tumour viruses in the study of EV biogenesis and trafficking [115, 116].

Cytokines and chemokines tend to use extracellular vesicles (EVs) as the medium to regulate cancer progression, besides trafficking. EVs, with its capacity to be a potent carrier for intercellular communication with ME, can strengthen pathophysiological functions of parental and recipient cells. Besides, they also manage to shoulder the responsibility of biological macromolecules like nucleic acids, lipids, and proteins [117]. EVs have a peculiarity about choosing a different path altogether for cytokine discharge than the standard ER/Golgi routes said to have been suggested [118]. EVs derive their advantage from several types of cells, which include highly aggressive cancer cells and embryonic stem (ES). That is why, in pathophysiological practices, EVs are considered major players as they aid in the growth and spread of cancer. Also, the unique nature of cancer cells, which discharge maximum microvesicles (MVs), than what their counterparts normally do, is linked to heightened invasiveness and their growth. Cancer cell growth and proliferation are dependent on modifications in the genetic material, and MVs can play a crucial role, particularly in oncogenes' spread intercellularly [27, 100].

**Figure 2:**
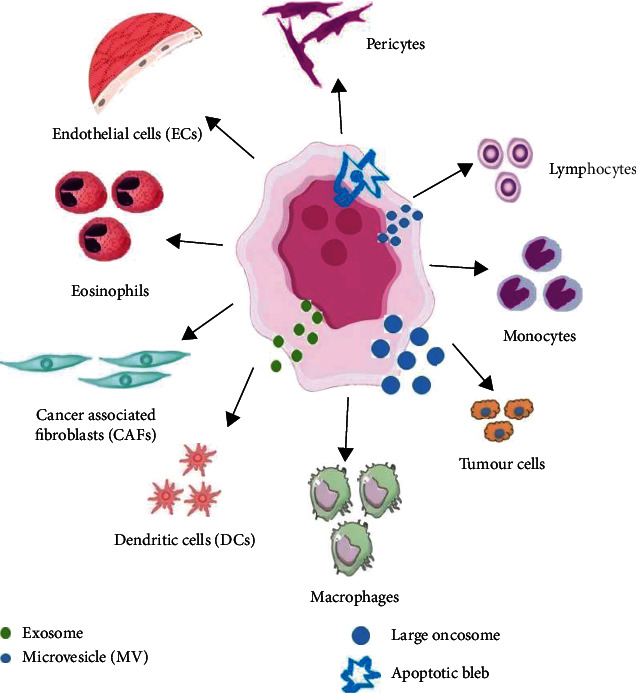
Tumour cells use EVs as the medium to converse with TME constituents. MVs, exosomes, and apoptotic bodies, which are the subgroups of EVs, have their roots, size, and structure, all based on biogenesis. MVs, referred to as vesicles (150-1000 nm), sprout straight from the plasma membrane. The term of exosome indicates to lesser vesicles (30-150 nm). Exosomes are responsible for the generation of intraluminal (ILVs) which, with inward invagination of endosome membranes, help growth of multivesicular bodies (MVBs). As for apoptotic bodies, they are known to be discharged during plasma membrane blebbing, particularly during programmed cell death [91].

EVs, being the constituent of an extended family consisting of membrane-bound vesicles, correspond to the third system of the intracellular contact system. They are in conjunction with cell-cell interactions and cytokines (soluble factors) mediated communication (Figure 2). The influence that cytokines wield over EV biogenesis and cargo has forced researchers into assuming various theories for loading cytokines into EVs. The crux of the hypothesis is that its entrapment might be to protect the cells from an autocrine outcome and, at the same time, discard the excessive products. Another possible explanation is that it shields the cytokines from environmental ruin [111]. EVs, in the company of cytokines, reach their final destination—the distant target cells and their absorption by cells that otherwise may not be possible when cytokines are present in solution. To understand the entire gamut of EV-linked cytokines a meticulous study is paramount. EVs have come to occupy a large canvas of pleiotropic functions, be it pathological or physiological conditions. Hence, the production of antigen presentation, sperm maturation, neuronal communication, and protection, besides acute and chronic inflammatory and autoimmune diseases or cancer, needs to be properly regulated [112]. More so, the moment for a new, not invasive therapeutic and diagnostic applications, has arrived because EV membrane composition, and its cargo is capable of reflecting the physiological conditions of its cells' origin which consequently inform about whether the cell is normal or damaged (tumour) [91, 100, 119].

**Figure 3:**
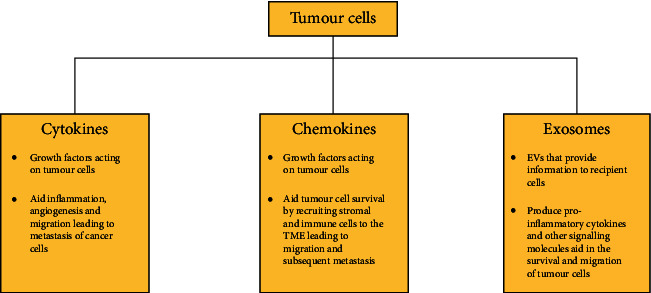
Tumour cells secrete cytokines, chemokines, and exosomes to mediate their progression, survival, and metastasis [91].

Induction of myeloid-derived suppressor cells instigates tumour growth. They do so by stalling the action of lymphocyte against IL-2 while favouring T cells. EVs originating from tumour consisting of TGF-*β* are involved in this process [105]. One study, during proteomic analysis, found exosome having proteins like VEGF, MCP-1, IL-4, and EGF—they aid tumour cells survival, growth, and transit—those exosomes with TGF-*β* stimulated division of fibroblasts into myofibroblasts, supporting tumour proliferation, vascularisation, and metastasis. The latter happened only when adequate TGF-*β* expression occurred on the surface of exosomes. Along with this, the exosomal surface witnessed the expression of the transmembrane proteoglycan *β*-glycan (Figure 3) [91, 120].

Ectosomes or microparticles, known for the biogenesis of MVs and chemokines MVs, are rather large than exosomes in size and, in different pathophysiological conditions, various types of MVs can appear [114]. “Large oncosome” which is cancer-derived EV population is outsized than other EV types known previously, according to a recent study. An aggressive amoeboid phenotype was noticeable in the biogenesis of huge oncosomes (Figure 2) [91, 100].

Some evidence proved TMVs prompt chemotaxis of leukocytes. NSCLC along with other adenocarcinomas (colorectal and pancreatic) *in vitro* cell lines discharge vesicles. Compared to nontriggered cells, TNF-*α* and CCL11 provoke MVs [121]. TMVs are capable of transferring some of the proteins and mRNA of tumour cells to monocytes, besides activating the latter. They can also change the biological functioning and immunologic phenotype of the recipients by inducing transfer of CD44v7/8 and CCR6 to monocytes through activation of serine/threonine kinase (AKT) [86]. Antiapoptotic effect on monocytes can be caused by TMVs that also stimulate Monocyte Chemoattractant Protein-1 (CCL-2), lens fibre-1*β* Major Intrinsic Protein (MIP-1*β*) (CCL4), IL-8 (CXCL-8), and Macrophage Inflammatory Protein-1*α* (CCL3) expressions. When activated, they can control secreted CCL-5 chemokines, normal T-cells expressed, and monocyte mRNA accumulation [122].

Killing of cancer cells and reverting drug resistance was the result of a study by Ma et al. According to it, drug-resistant tumour reproducing cells obtained from lung cancer-affected patients preferred cisplatin carrying MVs [41]. Cancer immunotherapy relies on the inherent immune response against the tumour [123]. The MVs, with their ability to express biological information and bioactive molecules, are gaining currency because immunotherapy involves supplying immunogens that are cancer-specific and kick-off cancer immunity mediated by T-cell. Conflicting interpretations on the prolificacy of TMVs is palpable because of the immune system, TMEs, and the complex cross-talk among cancer cells [91, 124].

MVs studies have, over a decade now, generated enthusiasm among researchers because of their contribution towards the pathogenesis of different kinds of cancers. The use of TMVs *in vivo* animal models or *in vitro* cell lines as diagnostic or predictive biomarkers has highlighted the significance of MVs as they are supposedly the means that surround cancer growth [125]. On the other hand, TMV involvement in chemoresistance and immune modulation of cancer cells cannot be ignored. All these prompt targeted remedial or applied TMV-based intercessions to broaden the benefits of immunotherapy or chemotherapy [27]. A genuine *in vivo* physiological job would entail designing animal models that can absorb or discharge chemokine comprising MVs to constantly monitor and intervene. The refreshing process of MVs, thanks to them being intercellular communication module, may well be viewed with greater anticipation in cancer biology (Figure 3) [27, 124].

## 5. Concluding Remarks

The one-size-fits-all in cancer therapy is a myth. The physicians broke it in the era of personalised medicine, guided by tools like genomic analysis and distinct molecular testing to ascertain fitting therapies. Even with advancements in precision surgery, chemotherapy, and radiotherapy, much progress with prognosis in the metastatic stage of solid tumours is not in sight. TME, owing to its heterogeneous composition, genetic/epigenetic mutations, tissue-specific responses, and variations in the genetic backgrounds of hosts, is the real culprit. Under the circumstances, the mainstay in most solid cancers, including lung surgical, remains surgical resection. Yet, postoperative complications, in many cases, have led to acute inflammation, attracting immune and mesenchymal cells to the resected location for wound healing. This is followed by a long haul of immune suppression, locally and systemically, increased progression and migration of residual cancer cells (CTCs), steering metastasis, known as a surgical-stress response. In the TME of NSCLC, the noncancerous cells (fibroblasts, macrophages, ECs, MSCs, and immune cells) are engaged by CTCs to aid tumour progression and metastasis. A huge number of cytokines, angiogenic, and growth factors and MMPs are produced by these cell types. Studies suggest that the malignant cells could affect the metabolism of stromal cells to turn them proliferative.

Tumour cells disrupt the cytokine/chemokine system, making them big players in regulating the TME. These small proteins, along with their receptors, activate and modulate certain signalling pathways, accelerating tumour growth. MVs, another key regulator of the TME, add to cancer pathogenesis. TMVs have shown to support the immunomodulation of cancer cells and chemoresistance. Immunotherapy targeting these immune checkpoints can provide a rationale to design newer drugs. Although substantial research has been conducted, involving certain cytokines/chemokines as prognostic biomarkers, a lot is left to be desired. Success in identifying targets in immune cells and angiogenesis has bolstered fervour in better understanding of TME's pathophysiological roles. For example, PD-L1, a successful prognostic biomarker for NSCLC therapy, does not qualify as a therapy for all patients. Therefore, it is imperative to identify additional biomarkers to increase the patient base. It is a fact that this strategy entails a huge cost and time. But using integrated system biology and computational modelling for better understanding of their crosstalk is a way out to recognise new therapeutics. Last, but not the least, an improved comprehension of cytokine/chemokine roles, their receptors and the signalling pathways active in metastasis would fund the existing therapy. It may also lead to better results when rational combinations with chemotherapy are undertaken. The emerging field of immunooncology, with sustained clinical and translational research, would provide information on these critical modulators and how they can be exploited for improved results.

## 6. Future Directions

Cytokines have proved to be successful in cancer treatment, but the impact of certain promising targets on different immune cell populations is still unknown. The collection and functions of surface receptors are reasonably well known for well-studied populations, such as T-cells, and researchers use roughly the same sets of CD markers to classify populations using flow cytometry. However, the less studied the immune cell population is, the more complex the sets of determined receptors are, and the more difficult it is to compare the findings to find persistent trends. The analysis of changes in the role and expression of surface markers of each individual immune system cell population after immunotherapy would simplify and unify the evaluation of therapy effectiveness, as well as allow for the prediction of immunotherapy effectiveness using surface markers of cancer patients' immune cells.

Despite the fact that the focus of this review is on a thorough overview of the prospects for cytokine-based cancer treatment, a review of the functions and surface markers of immune system cells in cancer and after cytokine-based immunotherapy is essential. The pleiotropic effect of cytokines as immune response regulators is one of their most notable characteristics. Each cytokine affects a variety of immune cell populations, allowing them to promote both antitumour and protumour responses. As a result, the development of combined schemes aimed at enhancing the antitumour response while suppressing immune cells that promote tumour growth would be critical for the future of cytokine-based cancer therapy. The short half-life and systemic toxicity (proinflammatory and autoimmune reactions) of high doses of cytokines, which are needed to induce a meaningful response in cancer patients, are also significant issues. To address the limitations of various cytokine therapies, new methods that enhance cytokine targeting and alter their pharmacokinetics (such as cell-based or other vector delivery and chemically modified recombinant proteins) may be useful. According to current trends in cancer immunotherapy, cytokines can play the most important role in treatment when used in conjunction with other drugs including immune checkpoint inhibitors, oncolytic viruses, or as a component of DC- and tumour cell-based vaccines.

## Figures and Tables

**Table 1:** Roles and functions of the cellular components secreting cytokines/chemokines in the tumour microenvironment.

Cell type	Function in TME	References
Tumour-associated macrophages (TAMs)^#^	TAMs exhibit M2 macrophage phenotype, i.e., protumorigenic in nature, anti-inflammatory, and secrete T_h_2 cytokines. Aid angiogenesis and invasion of cancer cells to secondary sites.	[126]
TIE-2-expressed macrophages (TEMs)	TEMs are the monocytes that express angiopoietin receptor, TIE-2. Engage in paracrine signalling with angiopoietin expressing endothelial cells aiding tumour angiogenesis.	[127]
Cancer-associated fibroblasts (CAFs)	Active stromal-cell populations, aid desmoplastic tumour niche. Promote angiogenesis and mediate tumour-promoting inflammation by releasing cytokines.	[29]
Neutrophils	N1-type possess antitumorigenic, proinflammatory and secrete T_h_1 cytokines. N2-type possess protumorigenic, anti-inflammatory and secrete T_h_2 cytokines.	[35]
Natural killer (NK) cells	Cytotoxic lymphocytes, in the absence of antigen presentation, kill stressed cells. Detect and kill tumour cells through “missing self” activation “stress-induced” activation.	[30]
Dendritic cells (DCs)	Antigen-presenting cells (APCs) that regulate adaptive immune response. In the TME, they promote angiogenesis by stimulated vascularisation.	[128]
Mast cells (MCs)	Generate, maintain innate and adaptive immune responses. Release factors that stimulate endothelial cell growth to promote angiogenesis in tumour cells.	[42]
Myeloid-derived suppressive cells (MDSCs)	Disrupt tumour immune surveillance by interfering with T and NK cells functions. Promote M2 macrophage polarisation.	[129]
B-cells	Modulators of humoral immunity, protumorigenic in nature and secrete cytokines. Alter T_h_1:T_h_2 ratio.	[33]
Regulatory T (T_reg_) cells	Suppress immune surveillance and elicit protumorigenic roles. Positively correlate with overall survival (OS) in multiple cancers.	[47]
CD4+ T_h_ cells	Separated into—T_h_1 and T_h_2 lineages. T_h_1 secrete proinflammatory cytokines and are antitumorigenic in nature while T_h_2 secrete protumorigenic cytokines and are anti-inflammatory.	[55]
CD8+ T_c_ cells	Effector cells of the adaptive immune system that recognise and destroy tumour cells via perforin-granzyme-mediated-apoptosis.	

^#^Macrophages have two subtypes based on the pathway they use for activation—classically activated M1 type and alternatively activated M2 type. Type M1 secretes T_h_1 cytokines and has proinflammatory and antitumorigenic roles while M2 type secretes T_h_2 cytokines and possesses anti-inflammatory and protumorigenic in nature. T_h_1: T_h_2 ratio possesses a correlation with tumour grade and stage [130].

**Table 2:** This table depicts cytokines, their source of production, and functions.

Cytokine	Source	Functions	References
IL-6	T-cells, macrophages, adipocytes	Proinflammatory action, differentiation, cytokine production	[5, 30, 80]
IL-8	Epithelial cells, macrophages, endothelial cells	Proinflammatory action, angiogenesis, chemotaxis	[5, 106]
IL-10	Monocytes, B-, and T-cells	Anti-inflammatory action, inhibition of proinflammatory cytokines	[63]
IL-17	T_h_ 17 cells	Proinflammatory action, cytokine and chemokine production, antitumour immunity	[87]
IL-27	Antigen-presenting cells (APCs)	Anti-inflammatory action, IL-10 production	[85]
IL-35	Regulatory T-cells (T_regs_)	Anti-inflammatory action induces proliferation of T_regs_ and suppresses T_h_ 17 cells	[60]
IL-37	NK cells, monocytes, epithelial cells, B-cells	Anti-inflammatory action, antimicrobial, antitumour immunity	[86]
TNF-*α*	Macrophages, CD4+ lymphocytes, adipocytes, NK cells	Proinflammatory action, cell proliferation, cytokine production, apoptosis	[26]
IFN-*γ*	NK cells, T-cells	Antiviral, proinflammatory action	[131]
TGF-*β*	T-cells, macrophages	Anti-inflammatory action, inhibition of proinflammatory cytokine production	[26, 52]
Granulocyte-macrophage colony-stimulating factor (GM-CSF)	T-cells, macrophages, fibroblasts	Proinflammatory action, improve neutrophil and monocyte function, macrophage activation	[30, 132]
Vascular endothelial growth factor (VEGF)	Macrophages, endothelial cells, platelets,	A growth factor that aids in vasculogenesis, angiogenesis, chemotaxis, migration of endothelial cells	[26]

**Table 3:** This table provides a brief of the major chemokines and their roles.

Chemokine	Functions	References
CCL-2	Recruits' monocytes, macrophages; aids in tumour progression, angiogenesis, metastasis	[48]
CCL-5	Recruits' neutrophils, T_regs_; aids survival of tumour cells by angiogenesis stimulation	[43]
CCL-18	Produced by monocytes, macrophages and immature DCs; proinflammatory, immune regulation; aids in tumour progression by mediating TME	[43]
CXCR-4	Promotes antitumoural T-cell responses	[122]
CX3CL-1	Helps in M2 macrophage polarisation; relocates monocytes, mast cells, T-cells, and NK cells to the site of inflammation	[17]
CXCL-1	Recruits' neutrophils, T_regs_; aids survival and proliferation of tumour cells	[133, 134]
CXCL-5	Leukocyte placement; aids tumour growth, angiogenesis, and metastasis	[103]
CXCL-8	Produced by macrophages; proinflammatory, recruits' neutrophils; aids survival and proliferation of tumour cells	[103]
CXCL-13	Produced by follicular DCs and T_h_ cells; infiltrates B-cells into tumour cells	[135]

## Data Availability

The data can be obtained from the corresponding author on request.
